# The Association between Mothers’ Smartphone Dependency and Preschoolers’ Problem Behavior and Emotional Intelligence

**DOI:** 10.3390/healthcare10020185

**Published:** 2022-01-18

**Authors:** Jeong-Soon Kim, Jungmi Kang, Hyunjung Lee

**Affiliations:** 1 Department of Nursing, Gwangju Health University, Gwangju 62287, Korea; jskim@ghu.ac.kr; 2 Department of Nursing, Dankook University, Cheonan 31116, Korea; jung88922@naver.com; 3 Department of Nursing, College of Nursing, Konyang University, Daejeon 35365, Korea

**Keywords:** preschoolers, mothers, smartphone dependency, problem behavior, emotional intelligence

## Abstract

This study aimed to identify the level of mothers’ smartphone dependency and determine its correlation with preschoolers’ problem behavior and emotional intelligence. From 1 November to 30 December 2020, 141 mothers of preschool children (aged three to six years) were recruited to complete questionnaires that assessed their smartphone dependency and their child’s problem behavior and emotional intelligence. The result revealed that the younger the mother and the higher the perception of boredom in daily living, the higher was the level of her smartphone dependency. Maternal smartphone dependency was also significantly correlated with the aggression, oppositional, and emotional instability subscales of the tool assessing children’s problem behavior. To prevent problem behaviors among preschoolers, strategies to reduce mothers’ smartphone dependency are needed.

## 1. Introduction

In today’s society, owing to the development of the internet-based service environment, smartphones have become an essential part of daily living that greatly impacts our lives. In South Korea, smartphone penetration has been constant at over 93% since 2017 [1]. Mothers not only discover practical parenting tips and information through smartphones but also engage in a variety of interactions with other parents without any temporal or spatial constraints [2].

Family is the first social environment that is experienced by a preschooler. In particular, the mother plays an important role and has the strongest influence on the formation of the basis of a child’s social development, as well as his or her attachment within the family. Therefore, a mother should sensitively respond to signs that her child manifests [3]. Preschool age signifies a period of the rapid growth of autonomy, independence, and an increase in physical activities. Any developmental issue that appears during this period can lead to characteristic behavioral patterns, such as tantrums and rejection. Parents may experience severe parenting stress and physical exhaustion while trying to tackle their children’s behavioral problems; this may act as an obstacle toward the formation of a stable parenting environment [3].

Not only can mothers relieve stress by interacting with other parents, but they can also receive counseling from childcare experts through smartphones [4,5]. However, excessive smartphone dependency can cause other problems, for example, mother–child attachment disorders, reduced parenting efficacy, maternal neglect, increased anxiety and aggression, social withdrawal, and smartphone addiction in children [4,6,7,8].

Preschooler’s problem behaviors are typically not expected and are difficult for teachers and parents to control [9]. Problem behaviors in children can be categorized into extrinsic behaviors that harm the child and those around them in the contexts of peer relationships, personal development, lifestyle, morality, emotional behavior, and intrinsic behaviors [9]. Harmful behaviors negatively affect the emotional and cognitive development of children, and if untreated, may permeate into adulthood, causing various psychological and social problems [9,10]. Children’s problem behaviors are greatly influenced by their disposition and their nurturing environments, such as home and childcare settings. Consequently, an increasing number of studies have reported that a mothers’ smartphone dependency triggers the emergence of problem behaviors in children [9,11,12]. The higher the level of parental smartphone dependency, the more passive their parenting behaviors are; therefore, children are more likely to be impatient and angry enough to grab their parents’ attention away from smartphones [13]. Young children are also likely to imitate their parents and become addicted to smartphones [4,8].

Emotional intelligence is the “ability to perceive and express emotion, assimilate emotion in thought, understand and reason with emotion, and regulate emotion in the self and others” [14]. Since this ability enhances self-awareness and self-regulation, it contributes to the maintenance of appropriate interpersonal relationships [15]. Children’s emotional intelligence is extremely important for the formulation of positive interactions with parents and peers. In parent–child interactions, parental reactions influence the development of their children’s nervous system [7]. A mother’s positive parenting attitude and stable emotional interactions help her child acquire emotional control skills and mature properly in the process of the development of emotional intelligence [16]. Such positive interactions are valuable experiences for children, and they are more likely to form good peer relationships and assimilate into a social group [16,17]. As examined above, preschool age is a significant period for the development of children’s emotional intelligence. If the mother is excessively dependent on her smartphone during this period, she tends to display fragmentary and unpredictable response patterns during mother–child interactions; this can heighten the child’s stress sensitivity and cause immature cognitive and emotional intelligence [7]. Therefore, children of parents with high levels of smartphone addiction or dependence tend to show negative developmental tendencies, such as impaired self-expression and self-regulation skills and increased aggression [18].

Consequently, this study aimed to identify the level of maternal smartphone dependency and its correlation with preschoolers’ problem behavior and emotional intelligence.

The purpose of this study was to investigate the levels of mothers’ smartphone dependency and their correlation with preschoolers’ problem behavior and emotional intelligence. Therefore, three concrete objectives were established:

1. Identify the level of smartphone dependency of mothers of preschool-age children and the level of problem behavior and emotional intelligence in preschoolers.
2. Determine the difference in preschoolers’ problem behavior and emotional intelligence based on the level of maternal smartphone dependency.
3. Verify the correlation between maternal smartphone dependency and preschoolers’ problem behavior and emotional intelligence.

## 2. Materials and Methods

### 2.1. Study Design

This is a descriptive correlational study that investigates the level of smartphone dependency among mothers of preschool-age children and tries to verify an association between maternal smartphone dependency and preschoolers’ problem behavior and emotional intelligence.

### 2.2. Participants

The participants of this study comprised mothers living in urban areas who (i) had preschool children aged 3–6 years, attending general early childhood education and care facilities without serious diseases or developmental disorders, (ii) were smartphone users, and (iii) voluntarily agreed to participate in the study after understanding its purpose. G * power 3.1.9 was used to determine the sample size [19]. The minimum acceptable sample size for the study was calculated at 132 participants (effect size = 0.3, significance level = 0.05, and power = 0.95). Considering a data dropout rate of 20%, the questionnaire was distributed to 159 mothers. After excluding 18 unretrieved or incomplete questionnaires, data from 141 subjects were used for analysis.

### 2.3. Psychometric Tools

#### 2.3.1. Maternal Smartphone Dependency

The level of maternal smartphone dependency was measured using the “Smartphone Dependency Scale” developed by Park [20] and modified/complemented by Yoon [21]. This 20-item scale comprises four 5-item subscales of morbid immersion, life interference, loss of control, and compulsive symptoms. Each item is rated on a 5-point scale, wherein a higher total score indicates a higher level of smartphone dependency. The internal consistency—defined by Cronbach’s α—was 0.95 in Yoon’s study [21] and 0.88 in this study.

#### 2.3.2. Problem Behavior

The children’s problem behavior was measured using the problem behavior assessment scale developed by Larzelere et al. [22] and modified/complemented by Kim [23]. This 53-item scale consists of the following five subscales: aggression (12 items), oppositional (10 items), emotional instability (11 items), immaturity (13 items), and shyness (7 items). Each item is rated on a 4-point scale, wherein a higher total score indicates a higher severity of problem behavior. Cronbach’s α in Kim’s study [23] and this study was 0.94 and 0.91, respectively.

#### 2.3.3. Emotional Intelligence

The children’s emotional intelligence was measured using the children’s emotional intelligence assessment scale developed by Mayer and Salovey [24] and modified/adapted by Lee [24] as a teacher’s checklist for emotional intelligence. This 31-item scale consists of the following four subscales: emotional self-awareness (7 items), self-emotion regulation (8 items), recognition of others’ emotions (7 items), and interpersonal emotion regulation (9 items). Each item is rated on a 5-point scale, wherein a higher total score indicates higher emotional intelligence. Cronbach’s α in Lee’s study [24] and this study was 0.89 and 0.90, respectively.

### 2.4. Ethical Considerations

After obtaining approval from the Institutional Review Board (IRB) of the affiliated university (IRB No. *** -2020-10-016), data were collected in accordance with the prior agreement and cooperation of the concerned facilities. Access to the collected data was limited to participating researchers, and all research-related documents were sealed and stored in a double-locked cabinet in the laboratory. The participants were assured of their anonymity and the data’s confidentiality, that the data would be strictly used for research purposes, and that it would be disposed of after a retention period of three years.

### 2.5. Data Collection

Data collection for this study was conducted from 1 November to 30 December 2020. The researcher visited two kindergartens (general education and childcare facilities) in C city, explained the study’s purpose and method to the director and staff, and obtained permission and consent to proceed with data collection. Based on the selection criteria, participants were recruited by sending a recruitment email to parents containing documents that explained the purpose of this study and participation methods, including the details of voluntary participation, anonymity, confidentiality, and possibility of withdrawal. After their confirmation, the questionnaire was sent to the participants, along with an IRB-approved informed consent form. They were requested to submit both documents on their next visit to the kindergarten. To avoid common method bias [25], children’s emotional intelligence was assessed by asking teachers. The teachers directly observed their behavior while interacting with them in classrooms or on weekdays, using a checklist developed as a teacher-reported assessment tool. A total of 11 teachers participated in this observational study. Credibility was established through the process of sample assessment, wherein all teachers discussed the data and reached a consensus. However, children’s problem behavior was assessed by their mothers. Children’s problem behaviors caused by the mother’s smartphone dependence are based on the assumption that these behaviors are manifested at home.

### 2.6. Data Analysis

Once collected, the data were analyzed using IBM SPSS (IBM Corp., Armonk, NY, USA) statistics for Windows, version 22.0. Statistical analyses included descriptive statistics. The *t*-test and one-way analysis of variance were used to analyze the differences in smartphone dependency, emotional intelligence, and problem behavior according to the general characteristics of mothers and children. Differences in the general characteristics based on maternal smartphone dependency were analyzed using the chi-square test, and differences in emotional intelligence and problem behavior were analyzed using the t-test. Correlation analysis was performed to determine the associations between maternal smartphone dependency and children’s emotional intelligence and problem behavior. Psychometric reliability was calculated using Cronbach’s alpha. The level of significance was set at *p* < 0.05.

## 3. Results

### 3.1. Level of Smartphone Dependency According to General Characteristics

The average age of the preschoolers who participated in this study was 4.75 ± 0.77 years, wherein girls outnumbered boys (62.4 vs. 37.6%). The average age of the mothers who participated was 36.95 ± 2.97 years, and most of them were in their late 30 s. Among them, 45.4% had a college degree or higher. The average time spent on internet use was 2.67 ± 1.33 h/d, and the most frequently cited reason for using the Internet was boredom (43.3%). Under this reason, the average smartphone dependency score for boredom was 49.18 ± 9.66 points, which was significantly higher than the score for stress relief and information acquisition on child-rearing (F = 9.02, *p* = < 0.001). Other characteristics did not significantly affect smartphone dependency (Table 1).

### 3.2. Mothers’ Smartphone Dependency and Preschoolers’ Problem Behavior and Emotional Intelligence

The overall average score for the mothers’ smartphone dependency was 45.28 ± 10.67. The average subscale scores were 12.11 ± 3.42 for morbid immersion, 10.99 ± 3.14 for life interference, 13.47 ± 3.41 for loss of control, and 8.70 ± 2.98 for compulsive symptoms.

The overall average problem behavior score was 75.31 ± 12.06 points. The average subscale scores were 16.71 ± 3.24 for aggression, 16.57 ± 3.64 for oppositional, 14.95 ± 2.99 for emotional instability, 15.91 ± 2.81 for immaturity, and 11.15 ± 2.48 for shyness.

The overall average emotional intelligence score was 116.44 ± 12.48. The average subscale scores were 27.67 ± 3.39 for emotional self-awareness, 28.06 ± 5.04 for self-emotion regulation, 25.80 ± 4.31 for recognition of others’ emotions, and 34.90 ± 4.59 for interpersonal emotion regulation (Table 2).

### 3.3. Differences in Preschoolers’ Problem Behavior and Emotional Intelligence According to the Level of Maternal Smartphone Dependency

To identify the preschoolers’ emotional intelligence and problem behavior according to the level of maternal smartphone dependency, the participating mothers were divided into low-level (lower 50% score) and high-level (higher 50% score) groups based on the overall mean maternal smartphone dependency. The average smartphone dependency score for the low-level group and high-level group was 36.94 ± 6.13 and 54.49 ± 5.99, respectively. The average problem behavior score of the low-level group was 73.04 ± 11.36, which was significantly lower than that of the high-level group (t = −2.39, *p* = 0.018). The average emotional intelligence score of the low-level group and high-level group was 117.62 ± 14.06 and 115.14 ± 10.43, respectively (Table 3).

### 3.4. Correlation between Mothers’ Smartphone Dependency and Children’s Problem Behavior and Emotional Intelligence

A positive correlation was observed between mothers’ smartphone dependency and children’s problem behavior (*r* = 0.25, *p* = 0.002). However, no significant correlations were observed between mothers’ smartphone dependency and children’s emotional intelligence (*r* = −0.10, *p* = 0.229) (Table 4).

## 4. Discussion

This study was conducted to identify the correlation between the level of mothers’ smartphone dependency and preschoolers’ problem behavior and emotional intelligence.

The results revealed that there is a positive correlation between mothers’ smartphone dependency and children’s problem behavior. This corresponds with an extant study according to which mothers’ excessive use of digital devices directly affects their interactions with their children and their problem behaviors [11]. In previous research, children’s problem behavior increased when parents used smartphones, which in turn led to parenting stress and parent–child attachment disorders [7,11]. This corresponds with the fact that this vicious circle eventually triggers the internalization of problem behavior, such as withdrawal, in children [11]. Parents with high smartphone dependency display passive parenting attitudes, which trigger problem behaviors in children, such as aggression and smartphone addiction [6,7,8,11]. Mothers’ indiscriminate use of smartphones while spending time with their children negatively impacts their children’s growth. Therefore, it is necessary to inform such mothers of the adverse effects of their smartphone dependency on their preschooler’s development and guide them to use smartphones efficiently. For example, community health center nurses and clinical nurses could be recruited to educate young mothers prone to smartphone dependency regarding the use of smartphones to help acquire relevant information for child-rearing, and self-help groups can be formed to help them reduce unnecessary smartphone use.

Further, no statistical correlation was observed between mothers’ smartphone dependency and the children’s emotional intelligence development. This is consistent with previous studies according to which mothers’ smartphone usage habits do not affect toddlers’ emotional intelligence [17,18]. However, it was contrary to one study, which found that parents’ excessive use of smartphones induces fragmentary and passive responses during their interactions with their children, thus heightening the children’s stress sensitivity and causing immature cognitive and emotional intelligence [7].

These contradictory results may be ascribable to different study participants and timings of assessment. Since this study was conducted during the restrictions imposed due to COVID-19, the environmental factors may have influenced the increase in smartphone usage. This is supported by a study on Czech adults under the age of 29 years. The study found that the COVID-19 pandemic caused a considerable increase in the use of the Internet for tutorials of health-related activities [26].

Technoference is a neologism, a portmanteau of “technology” and “interference,” which signifies interrupted interactions and interpersonal relationships due to the use of digital devices such as smartphones. Technoference caused by parental smartphone dependency can negatively affect the child’s self-esteem formation and socio-emotional development; as such, parents cannot serve as appropriate role models for the formulation of correct human relationships using proper interactions [17]. In this context, many studies have argued that there is a direct or indirect correlation between parental smartphone dependency and children’s emotional intelligence development [11,12,17,27]. Therefore, to accurately determine the causal link between mothers’ smartphone dependency and children’s emotional intelligence and the extent to which the former influences the latter, it is necessary to accumulate data through multicenter studies and continuous repetition of studies with more participants.

In this study, it was also found that a high proportion of mothers used smartphones to relieve stress or boredom in addition to obtaining information regarding child-rearing. This corresponds with a previous study [27], according to which parents’ excessive use of digital devices is primarily due to anxiety and difficulty in controlling their emotions. Parents with young children use mobile devices as a means of emotional regulation in the contexts of cognitive tension between work and childcare, stress-induced emotional tension, and environmental tension threatening their daily living [27]. Consequently, such parents should be provided with various alternatives for parenting stress relief.

Smartphone health apps seem to reduce parenting-related costs and provide emotional support for parents during childcare, such as reducing healthcare costs, improving relationships with medical staff, reducing overdiagnosis, and efficient medical consultation [2]. In the age of the fourth Internet-based industrial revolution, smartphones are an indispensable tool for daily living, and their use is likely to expand further; this highlights the need to pay more attention to childcare-related smartphone app development and use. Therefore, instead of focusing only on the negative consequences of mothers’ smartphone dependency, efforts should be directed toward determining the reasons for their dependency. The results may be used for setting up various supportive measures for reducing the tension and stress arising due to the parenting process.

The finding of this study cannot be generalized because the participants were restricted to mothers of preschool-age children in one urban city. Moreover, mothers’ smartphone dependency and their children’s problem behavior were based on mothers’ reports. Therefore, we could not eliminate the fake effects.

Despite these limitations, this study is significant because it serves as a basis for developing strategies to help mothers with high levels of smartphone dependency become more aware of appropriate smartphone usage, foster children’s healthy development, and reduce smartphone dependency.

## 5. Conclusions

This study showed that the younger the mother and the higher the level of her perceived boredom, the higher the level of her smartphone dependency. Further, maternal smartphone dependency is significantly correlated with aggression, oppositional, and emotional instability among the subscales of children’s problem behavior.

Therefore, mothers must recognize the problem of indiscriminate smartphone use while spending time with their children to prevent the occurrence of problem behavior in their preschool-age children. In addition, it is necessary to formulate strategies to reduce indiscriminate smartphone use by mothers who raise preschool-age children and help them use smartphones more efficiently. In this context, this study presents the following research proposals based on the results.

First, a qualitative study in the future would help understand childcare-related experiences of preschool-age children’s mothers, who have a high level of smartphone dependency. Second, future studies should consider developing strategies to reduce smartphone dependency among mothers of preschool-age children and encourage the efficient use of smartphones.

## Figures and Tables

**Table 1:** Smartphone dependency according to general characteristics. (*n* = 141).

Variables				Smartphone Dependency	
		Categories	*n*(%)/M ± SD	M(SD)	t/F (*p*)
Child	Age (year)	3–4	75 (53.2)	44.70 (10.94)	−0.68 (0.496)
		5–6	66 (42.6)	45.93 (10.40)	
		Average	4.75 ± 0.77		
	Sex	Male	53 (37.6)	46.11 (10.08)	0.71 (0.476)
		Female	88 (62.4)	44.78 (11.03)	
Mother	Age (year)	≤35	46 (32.6)	48.26 (9.97)	6.49 (0.039)
		36–40	79 (56.0)	44.17 (11.21)	
		>40	16 (11.4)	42.18 (8.15)	
		Average	36.95 ± 2.97		
	Education	≤College graduation	50 (35.5)	45.22 (10.59)	0.12 (0.941)
		University graduation	64 (45.4)	45.57 (11.73)	
		>University graduation	27 (19.1)	44.70 (8.022)	
	Job	Yes	75 (53.2)	45.62 (10.25)	0.42 (0.669)
		No	66 (46.8)	44.84 (11.27)	
	Reason of use	Obtain information ^a^	53 (37.6)	41.15 (10.41)	9.02 (<0.001) a, b < c ^†^
		Stress ^b^	27 (19.1)	44.59 (10.47)	
		Bored ^c^	61 (43.3)	49.18 (9.66)	
	Time of use (hour/day)	Average	2.67 ± 1.33		

^†^ post hoc (Scheffe test).

**Table 2:** Mother’s smartphone dependency and preschooler’s problem behavior and emotional intelligence. (*n* = 141).

Variables	M ± SD	Min–Max	Skewness	Kurtosis
Smartphone dependency	45.28 ± 10.67	20–72	0.028	−0.476
Morbid immersion	12.11 ± 3.42	6–22		
Life interference	10.99 ± 3.14	5–18		
Loss of control	13.47 ± 3.41	5–21		
Compulsive symptoms	8.70 ± 2.98	4–16		
Problem behavior	75.31 ± 12.06	55–113	0.851	0.366
Aggression	16.71 ± 3.24	12–29		
Oppositional	16.57 ± 3.64	10–32		
Emotional instability	14.95 ± 2.99	11–23		
Immaturity	15.91 ± 2.81	13–28		
Shyness	11.15 ± 2.48	7–18		
Emotional intelligence	116.44 ± 12.48	80–147	0.171	0.163
Emotional self-awareness	27.67 ± 3.39	18–35		
Self-emotion regulation	28.06 ± 5.04	15–40		
Recognition of others’ emotions	25.80 ± 4.31	15–35		
Interpersonal emotion regulation	34.90 ± 4.59	24–45		

**Table 3:** Differences in children’s problem behavior and emotional intelligence according to the level of mothers’ smartphone dependency. (*n* = 141).

	Smartphone Dependency		t	*p*
	Low-Level Group (*n* = 74)	High-Level Group (*n* = 67)		
	M ± SD	M ± SD		
Problem behavior	73.04 ± 11.36	77.83 ± 12.40	−2.39	0.018
Emotional intelligence	117.62 ± 14.06	115.14 ± 10.43	1.19	0.235

**Table 4:** Correlation between mothers’ smartphone dependency and children’s problem behavior and emotional intelligence. (*n* = 141).

Variables	Smartphone Dependency	Problem Behaviors	Emotional Intelligence
	r(p)	r(p)	r(p)
Smartphone dependency	1		
Problem behaviors	0.25 (0.002)	1	
Emotional intelligence	−0.10 (0.229)	−0.15 (0.076)	1

## Data Availability

The data presented in this study are available on request from the corresponding author. The data are not publicly available due to privacy or ethical restrictions
